# A Rare Modality of Concurrent Cryptococcal and Tubercular Meningitis in a Patient Living With HIV

**DOI:** 10.7759/cureus.66032

**Published:** 2024-08-02

**Authors:** Madhulika L Mahashabde, Yash R Bhimani, Harin M Bhavsar, Jugal Sriram

**Affiliations:** 1 General Medicine, Dr. D. Y. Patil Medical College, Hospital and Research Centre, Dr. D. Y. Patil Vidyapeeth, Pune, IND

**Keywords:** opportunistic infection, cm/tbm co-infection, patient living with hiv, cryptococcal meningitis (cm), tubercular meningitis (tbm)

## Abstract

Patients with Human Immunodeficiency Virus/Acquired Immunodeficiency Syndrome (HIV/AIDS) and a low CD4 count have decreased humoral and cellular immunity, predisposing them to opportunistic infections. Opportunistic infections are one of the main causes of morbidity and mortality in immunocompromised individuals due to impaired immune systems, particularly in persons living with HIV/AIDS. Common opportunistic infections in patients living with HIV include bacterial infections such as Mycobacterium tuberculosis and Mycobacterium avium complex (MAC); viral infections such as cytomegalovirus (CMV) and herpes simplex virus 1 (HSV-1); fungal infections such as Pneumocystis carinii pneumonia (PCP) and cryptococcal meningitis; and parasitic infections such as cryptosporidiosis and toxoplasmosis. Concurrent infection with cryptococcal and tubercular meningitis in patients with HIV is very rare. Here, we present the case of a 48-year-old male living with HIV who presented with complaints of breathlessness, fever, and weight loss and was evaluated and put on antitubercular medications for pulmonary tuberculosis. However, the presence of a continuous headache led us to investigate further. Upon brain imaging and cerebrospinal fluid evaluation, it was determined to be meningitis due to co-infection with Mycobacterium tuberculosis and Cryptococcus neoformans. The patient was treated with antitubercular therapy along with antifungal therapy. He is under regular follow-up without any further events.

## Introduction

When CD4+ T-cell counts decline, people with chronic HIV infection who are not receiving antiretroviral therapy become susceptible to a variety of infections that are uncommon in immunocompetent hosts-hence the term "opportunistic infections" (OIs) [1].

In individuals with advanced HIV disease and CD4 ≤ 100/µL in 2014 [2], cryptococcal infection accounted for 15% of all causes of death; by 2020, it constituted 19% of mortality in those with CD4 < 200 cells/µL [3]. Cryptococcal meningitis (CM) is the most common cause of meningitis in patients living with HIV [4].

Pulmonary tuberculosis is very common in patients with HIV. Tuberculous meningitis (TBM) is a severe form of extrapulmonary tuberculosis (EPTB) associated with a high rate of morbidity and mortality [5]. The most common cause of death for HIV-positive individuals is TBM and its associated consequences, such as vasculitic infarct with hemiparesis and elevated intracranial tension leading to obstructive hydrocephalus.

There are very few cases of coinfection with TBM and CM in the literature in patients with HIV. The diagnosis of CM/TBM coinfection is difficult due to the paucibacillary nature of the tubercular component [6]. We report an unusual case of CM/TBM coinfection in a patient living with HIV, diagnosed early and treated successfully.

## Case presentation

A 46-year-old man, diagnosed with Acquired Immunodeficiency Syndrome (AIDS) three months ago, was on antiretroviral therapy consisting of Lamivudine, Tenofovir, and Dolutegravir, and had no known comorbidities. The patient presented to us with complaints of weight loss of approximately 6 kg over the past three months, along with loss of appetite. He has had a low-grade intermittent fever for the past month, with an evening rise in temperature. He also reported breathlessness, which has progressed from MMRC grade II to grade III over the past month, cough with scant expectoration for the past 20 days, and a mild holocranial headache of subacute onset for the past 10 days. There was no significant past medical history, and the patient denies engaging in high-risk sexual behavior. He did report a history of blood transfusion five years ago.

On general examination, the patient appeared cachectic but conscious and oriented to time, place, and person. His pulse rate was 110 beats/min with normal blood pressure and a respiratory rate of 18 breaths/min. Oxygen saturation was 96% on room air. Pallor was noted, but there were no signs of icterus, cyanosis, clubbing, or lymphadenopathy. Upon admission, routine laboratory investigations are outlined in Table 1.

**Table 1:** Laboratory investigations on admission. Alanine transaminase (ALT), Aspartate transaminase (AST), and Alkaline phosphatase (ALP) are enzymes that assess liver function. Total leukocyte count (TLC) measures the number of white blood cells in the blood. Erythrocyte sedimentation rate (ESR) and C-reactive protein (CRP) are markers of inflammation. HbA1c: glycated Hemoglobin; Units of measurement include gram per deciliter (g/dL), milligram per deciliter (mg/dL), microgram per deciliter (µg/dL), nanograms per milliliter (ng/mL), cubic millimeter (cu.mm) and international units per liter (IU/L).

Parameters (units)	Laboratory values	Normal values
Hemoglobin (g/dl)	9.8 g/dL	13.2-16.6 g/dL
Total leukocyte count (cells/cu.mm)	5300/cu.mm	4000-10000/cu.mm
Platelet count (cells×10^6^/μL)	217000×10^6^/μL	4.35-5.65×10^6^/μL
Serum urea (mg/dL)	33 mg/dL	17-49 mg/dL
Serum creatinine (mg/dL)	0.86 mg/dL	0.6-1.35 mg/dL
Total serum bilirubin (mg/dL)	0.32 mg/dL	0.22-1.20 mg/dL
Direct bilirubin (mg/dL)	0.17 mg/dL	<0.5 mg/dL
AST (IU/L)	50 IU/L	8-48 IU/L
ALT (IU/L)	76 IU/L	7-55 IU/L
ALP (IU/L)	89 IU/L	40-129 IU/L
HbA1c(%)	5.5%	<5.7%
Total protein (g/dL)	7.5 g/dL	6.4-8.3 g/dL
Serum albumin (g/dL)	3.5 g/dL	3.5-5.2 g/dL
Albumin: globulin ratio	0.87	1.1-2.5
ESR (mm/hr)	25 mm/hr	<20 mm/hr
CRP mg/dL	2 mg/dL	<0.3 mg/dL
HIV antibody	Positive	
CD 4+ T cell counts	175/cu.mm	500-1500/cu.mm
Hepatitis B antibody	Negative	
Anti-hepatitis C antibody	Negative	

**Figure 1:**
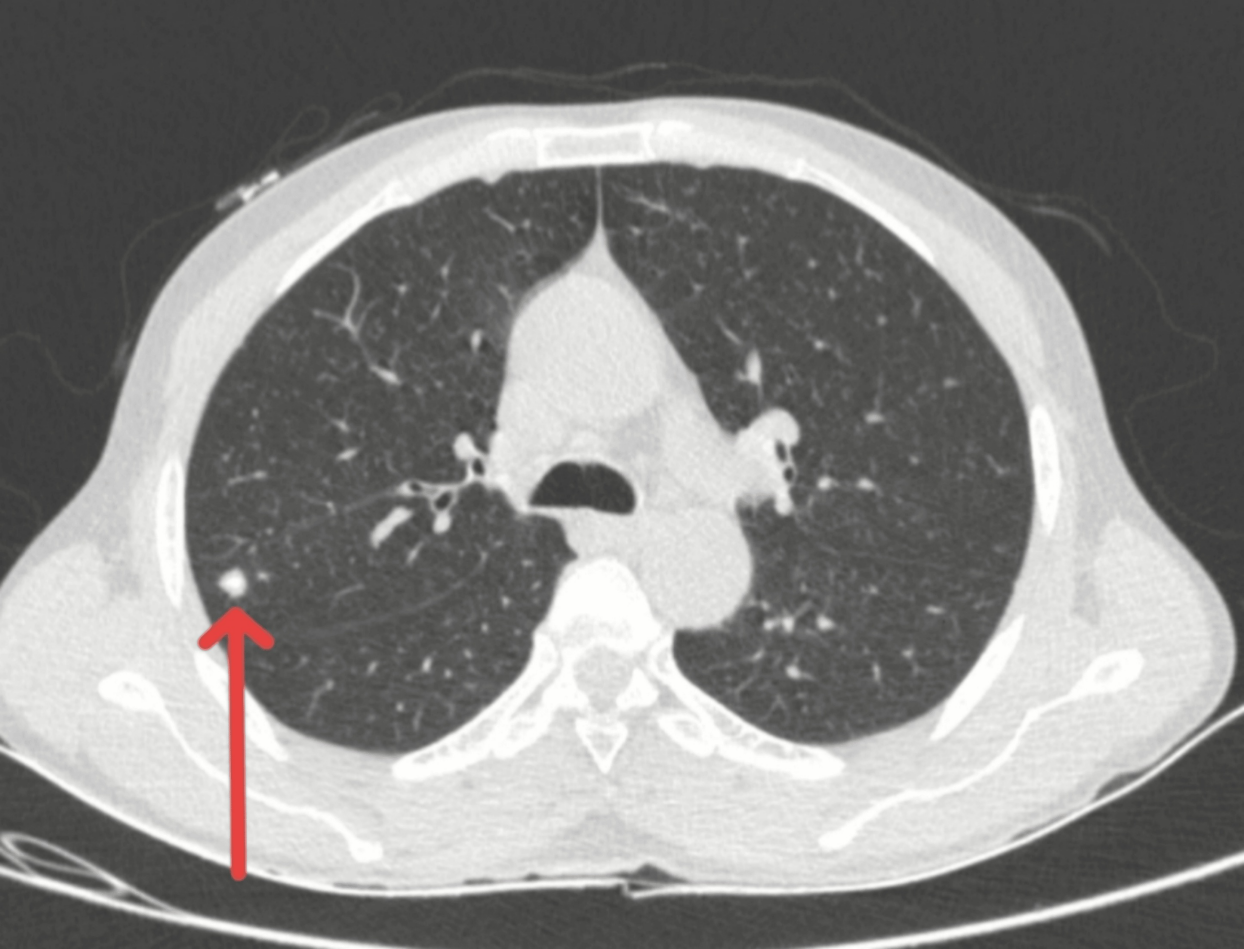
HRCT of the thorax showing centrilobular nodules and nodular opacities. HRCT: High-Resolution Computed Tomography

The chest X-ray showed branching linear and nodular opacities. High-resolution computed tomography (HRCT) of the chest with contrast was performed to evaluate for pulmonary tuberculosis. HRCT of the thorax revealed centrilobular nodules with nodular opacities (tree-in-bud appearance), as shown in Figure 1.

Sputum was tested using the Cartridge-based Nucleic Acid Amplification Test, which returned positive for pulmonary tuberculosis. The patient was initiated on anti-tubercular therapy with an intensive phase regimen containing Isoniazid, Rifampicin, Pyrazinamide, and Ethambutol adjusted for weight. The patient was put on tablet Trimethoprim-Sulfamethoxazole once daily for prophylaxis against Pneumocystis jirovecii pneumonia due to low CD4 cell counts.

**Figure 2:**
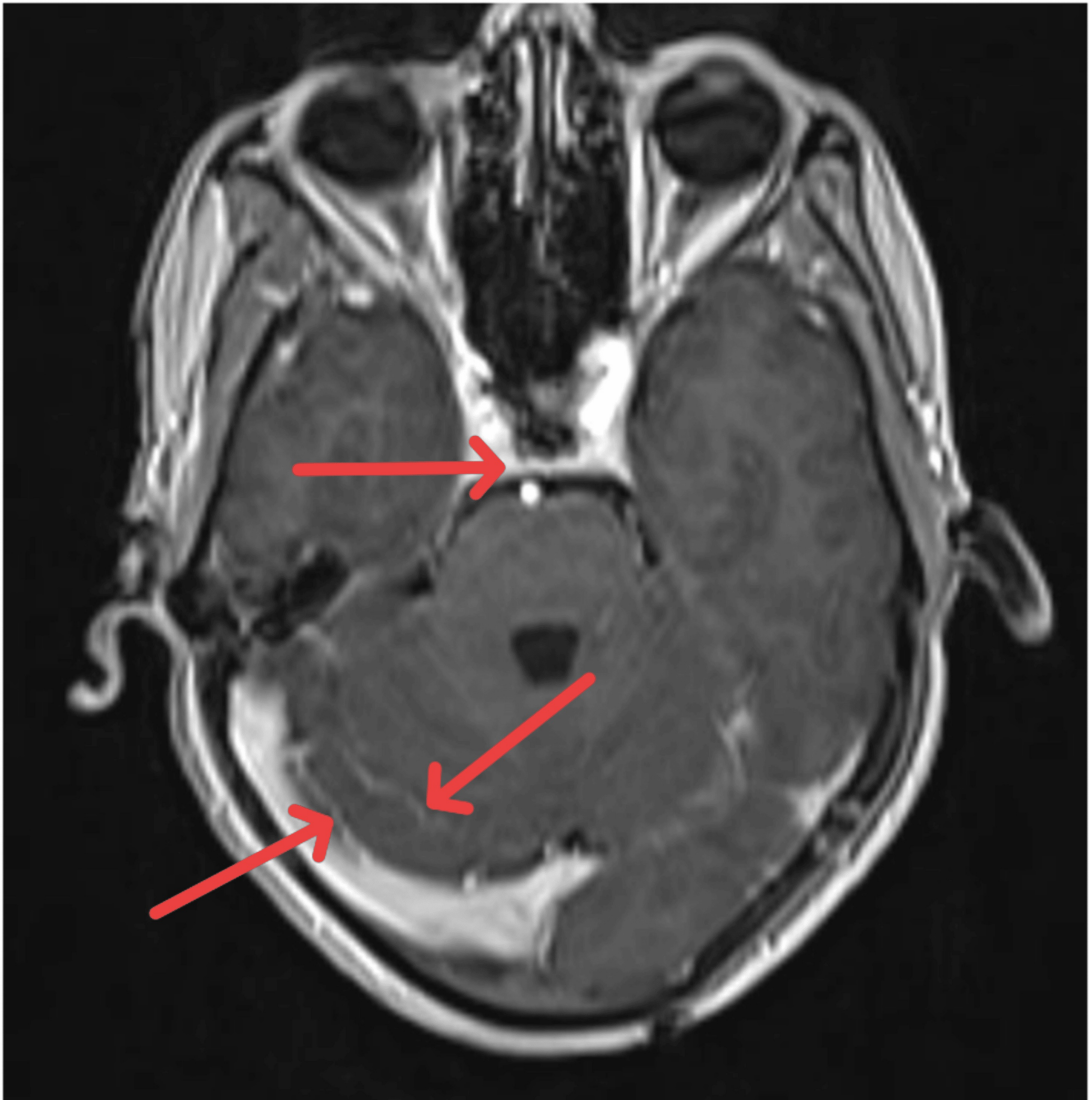
MRI brain contrast with veno-arteriogram: diffuse post-contrast enhancement along bilateral sulci, cistern spaces, and cerebellar folia (indicated by red arrow)

The fever subsided over five days, but the patient continued to experience headaches. The headache intensified to a moderate level, accompanied by neck rigidity. On the seventh day of admission, the patient experienced one episode of focal seizure. MRI of the brain with contrast and MR veno-angiography was performed to assess for tubercular meningitis. The imaging revealed diffuse post-contrast enhancement along bilateral sulci, cistern spaces, and cerebellar foliae, suggesting meningitis (as shown in Figure 2). Mild dilatation of the bilateral lateral and third ventricles was also observed.

Lumbar puncture was performed, and cerebrospinal fluid was sent for routine microscopic investigation and CB-NAAT (as shown in Table 2).

**Table 2:** Cerebrospinal fluid (CSF) analysis RBC: red blood cells; WBC: white blood cells; LDH: lactate dehydrogenase; ADA: adenosine deaminase; CSF: cerebrospinal fluid; CB-NAAT: cartridge-based nucleic acid amplification test; mg/dL: milligram/desi liter; cu.mm: cubic millimeter; U/L: unit/liter.

CSF routine microscopy parameters (units)	Laboratory values	Normal values
Appearance	Clear, transparent	Clear
Cob web	Absent	Absent
Glucose	25 mg/dL	40-80 mg/dL
Proteins	70 mg/dL	15-45 mg/dL
RBC	Absent	Absent
WBC	50/ cu.mm	0-5/ cu.mm
Neutrophils	10%	-
Lymphocytes	90%	-
LDH	132 U/L	<70 U/L
ADA	66 U/L	0-5 U/L
CSF CB-NAAT	Positive	Negative
CSF Culture	Cream-colored colonies with Budding yeast cells	
India ink preparation	Positive for Cryptococcus	

**Figure 3:**
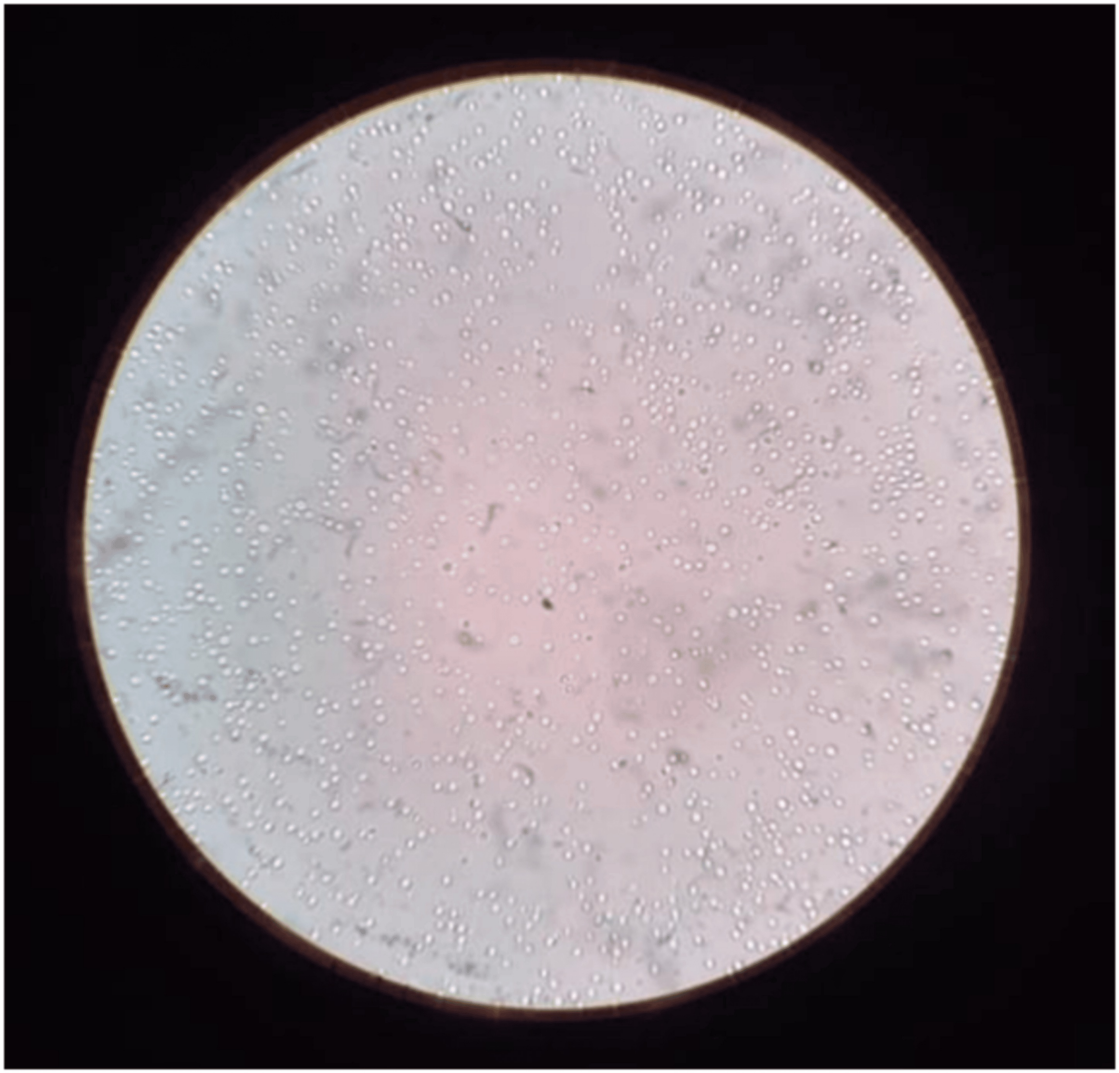
Negative staining with an India ink preparation showing Cryptococcus neoformans.

**Figure 4:**
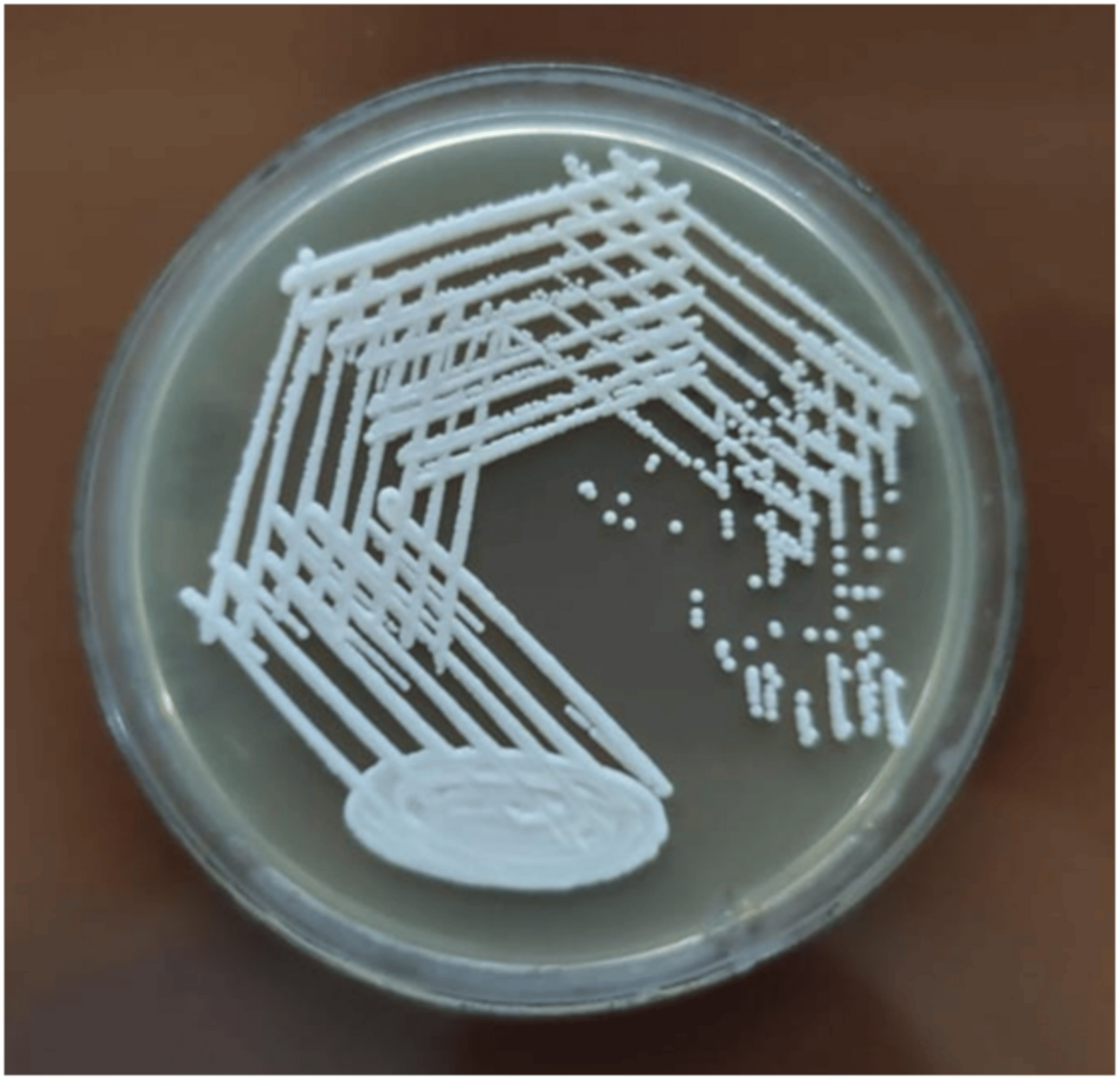
Sabouraud Dextrose Agar (SDA) showing growth of Cryptococcus neoformans.

Given the patient's immunosuppressive state from HIV/AIDS, negative staining with an India ink preparation and a culture on Sabouraud Dextrose Agar (SDA) were performed to rule out any other opportunistic infections. The India ink preparation tested positive for Cryptococcus neoformans (as shown in Figure 3). The organism grew on Sabouraud Dextrose Agar (SDA) (as shown in Figure 4).

The patient was administered Injection Amphotericin B deoxycholate at 0.7 mg/kg IV once daily, along with tablet Flucytosine at 100 mg/kg/day orally in four divided doses for two weeks, as the patient could not afford Injection Liposomal Amphotericin B. Following the induction phase, the patient received tablet Fluconazole 400 mg daily for ten weeks as consolidation therapy. Subsequently, the patient was placed on tablet Fluconazole 200 mg daily as maintenance therapy.

Injection Levetiracetam was initiated due to focal seizures, and anti-tubercular therapy was continued. Antiretroviral therapy, comprising Lamivudine, Tenofovir, and Dolutegravir, was resumed two weeks after starting antifungal therapy to avoid Immune Reconstitution Inflammatory Syndrome (IRIS). The patient is currently under regular follow-up without any further events.

## Discussion

HIV damages the immune system, increasing the risk of developing acquired immunodeficiency syndrome (AIDS), a disorder that makes a person more vulnerable to infections. With an estimated 39 million people living with HIV/AIDS worldwide in 2022, HIV/AIDS remains a global public health concern [7].

One of the main causes of death for patients with HIV/AIDS is tuberculosis (TB), and 99% of these deaths occur in resource-limited settings [8]. According to a study by Pandey et al. (2021) in Mumbai, the six-month mortality rate for patients with HIV-associated tuberculous meningitis (TBM) was as high as 45% [9]. Similarly, cryptococcosis is a devastating complication for patients with HIV/AIDS, accounting for approximately 55% of deaths in underdeveloped areas [10].

CM/TBM co-infection reflects the advanced immunosuppression characteristic of patients with HIV. Research has demonstrated that TB and fungal infections share many similarities in pathogenic processes and have a synergistic growth-promoting relationship [11].

Clinical presentations of TBM/CM co-infection in a patient with HIV include weakness, weight loss, cough, sputum production, chest pain, headache, and papilledema [12]. Due to the notable similarities in clinical presentation between TBM and CM, diagnosis may be missed or delayed. In our case, the patient presented with signs and symptoms of pulmonary tuberculosis. However, a thorough examination revealed the presence of a cryptococcal infection alongside central nervous system involvement of primary pulmonary tuberculosis. A concurrent diagnosis of TBM may not be made in CM patients due to the high early mortality rate associated with cryptococcal meningitis, which could lead to underdiagnosis of co-infection [6]. CM or TBM mono-infection cannot be distinguished from co-infection using any discriminating biomarkers as of yet.

With the advent of the new Xpert MTB/RIF generation (Xpert MTB/RIF Ultra), the minimal detection level has increased tenfold, from 100 CFU/ml to approximately 10 CFU/ml, resulting in a sensitivity of up to 70%, which is comparable to TB bacterial culture [13]. Currently, the WHO recommends GeneXpert MTB/RIF as the initial molecular diagnostic test for patients suspected of having TBM. In HIV-positive patients, cryptococcal meningitis can present as a very subacute, indolent illness, requiring a high degree of suspicion. Diagnosis is typically made using cerebrospinal fluid (CSF) culture, the cryptococcal antigen test (CrAg) in CSF/serum, and examination with India ink preparation. Quantitative cerebrospinal fluid (CSF) culture is an effective method for monitoring antifungal therapy during the follow-up period for cryptococcal meningitis. This test quantifies the number of yeast cells in the CSF, providing a measure of fungal burden and early fungicidal activity (EFA) during treatment. Regular CSF cultures allow for the assessment of fungal load reduction, thereby indicating the efficacy of the antifungal therapy [14].

According to current recommendations, antiretroviral therapy (ART) should be initiated in all patients with HIV-TB, regardless of their blood CD4+ cell count. A four-drug anti-TB regimen consisting of isoniazid, rifampin, ethambutol, and pyrazinamide should also be administered for TB [15]. In patients with an immunocompromised state such as AIDS and cryptococcal meningitis (CM), injection liposomal amphotericin B at 0.7 mg/kg/day IV, along with oral flucytosine at 100 mg/kg/day in four divided doses at six-hour intervals, is recommended. Following successful induction therapy, which results in clinical improvement and a negative CSF culture after two weeks, consolidation therapy should commence with oral or IV fluconazole at 400 mg once daily for ten weeks, followed by 200 mg daily for chronic maintenance for a minimum of one year [16]. Delaying the initiation of ART for at least two weeks after amphotericin B treatment may reduce the risk of Immune Reconstitution Inflammatory Syndrome (IRIS) [17].

## Conclusions

Cryptococcal meningitis concurrent with tubercular meningitis is a highly uncommon occurrence and poses significant diagnostic challenges. Individuals living with HIV are at a heightened risk of developing such rare infections, especially those with a CD4 count below 200. When assessing patients with compromised immune systems, physicians should maintain a high index of suspicion for diagnosing such opportunistic infections. Cryptococcal meningitis and tubercular meningitis impose a substantial disease burden on HIV patients if left untreated. Therefore, early diagnosis and prompt treatment are crucial to mitigate serious outcomes.
